# Supplementary material for: HiBC: a publicly available collection of bacterial strains isolated from the human gut
Source: Nat Commun. 2025 May 6;16:4203. doi: 10.1038/s41467-025-59229-9 (PMC12056005; doi:10.1038/s41467-025-59229-9)
Supplement: Supplementary file 1 — Supplementary Information [file 41467_2025_59229_MOESM1_ESM.pdf]

Supplementary Methods

Culture media

All quantities are given per liter of medium.

General process for agar plates: The components stated in the list below were supplemented with 15.0 g agar and a redox potential indicator (1.0 mg resazurin or 2.5 mg phenolsafranine). Afterwards they were dissolved in distilled water (99% of the final volume) and autoclaved (121°C, 15 min). A 100x concentrated stock solution containing heat sensitive components together with the reducing agents L-cysteine (final concentration 0.05% w/v) and DTT (final concentration 0.02% w/v) was created in distilled water and filter sterilized. All following steps were conducted in a laminar flow cabinet. After the autoclaved part of the media cooled down to approximately 60°C, the filtered stock solution was added (1% of the final volume). The still liquid agar was poured directly into petri dishes or one-well plates (Nunc™ OmniTray, Cat. No.: 140156). For the 96-well plates used in the case of single-cell dispensins, a multichannel pipette was used to transfer 250 µl of the medium into each well. The solidified medium was stored at 4°C and oxygen was removed by exposing the plates to an anaerobic environment for 48 h before use.

General process for Hungate tubes: The components stated in the list below were supplemented with a redox potential indicator (1.0 mg resazurin or 2.5 mg phenolsafranine) and the reducing agents L-cysteine (500 mg) and DTT (200 mg). After dissolving all components in distilled water to the final volume the medium was heated in a microwave until boiling and aliquoted into clean Hungate tubes (9 ml each). Oxygen was removed by submerging a gassing needle to the bottom of the Hungate and perfusing the hot medium with a forming gas/CO_2 mixture (89.3% N_2, 6% CO_2, 4.7% H_2) for 3 min. While removing the needle the empty part of the Hungate tube was flushed with the gas mixture as well and immediately sealed with a rubber stopper and a screw cap. Finally the Hungate tubes were autoclaved (121°C, 15 min) and stored at room temperature until use.

List of media:

BBE (Bacteroides Bile Esculin with amikacin): (BD, ref. PA-254480.02) pancreatic digest of casein, 14.5 g; papaic digest of soybean meal, 5 g; NaCL, 5 g; esculin, 1 g; ferric ammonium citrate, 0.5 g; oxgall, 15 g; hemin, 0.01 g; amikacin 0.075 g; vitamin K1, 0.01 g; growth factors 1.8 g.

BHI (Brain-heart-infusion): (DSM Medium 215c) BHI (Oxoid, ref. CM1135B), 37 g.

GMM (Gut Microbiota Medium): tryptone peptone, 2 g; yeast extract, 1 g; D-glucose, 0.4 g; L-cystein, 0.5 g; cellobiose, 1 g; maltose, 1 g; fructose, 1 g; meat extract, 5 g; KH_2PO_4, 13.6 g; $MgSO_4$ x 7 H_2O, 0.002 g; $NaHCO_3$, 0.4 g; NaCl, 0.08 g; $CaCl_2$, 0.008 g; vitamin K (menadione), 0.001 g; $FeSO_2$, 0.4 mg; hematin, 1.2 mg solved in 0.2M histidine; Tween80, 0.05 % v/v; ATCC Trace Mineral Mix, 10 ml; acetic acid, 1.7 ml;

isovaleric acid, 0.1 ml; propionic acid, 2 ml; butyric acid, 2 ml. Heat sensitive components (added after autoclaving): ATCC Vitamin Mix, 10 ml.

mGAM (modified Gifu Anaerobic Medium): (DSM Medium 1715) GAM Agar, Modified (HyServe, ref. 05433), 41.7g.

mGAM blood: mGAM as described above with addition of defibrinated sheep blood, 50 ml. The sheep blood was added after autoclaving (without filter sterilization) and only used for the creation of agar plates.

WCA (Wilkins-Chalgren Anaerobe broth): (DSM Medium 339a) WCA (Oxoid, ref. CM0643), 33 g.

YCFA (Yeast Casitone Fatty Acids): (DSM Medium 1611) Casitone, 10.0 g; yeast extract, 2.5 g; glucose, 5.0 g; $MgSO_4$ x 7 H_2O, 0.045 g; $CaCl_2$ x 2 H_2O, 0.09 g; K_2HPO_4, 0.45 g; KH_2PO_4, 0.45 g; NaCl, 0.9 g; $NaHCO_3$, 4.0 g; hemin, 0.01 g; acetic acid, 1.9 ml; propionic acid, 0.7 ml; isobutyric acid, 90.0 µl; n-valeric acid, 100.0 µl; iso-valeric acid, 100.0 µl. Heat sensitive components (added after autoclaving): Biotin, 0.02 mg; folic acid, 0.02 mg; pyridoxine-HCl, 0.1 mg; thiamine-HCl x 2 H_2O, 0.05 mg; riboflavin, 0.05 mg; nicotinic acid, 0.05 mg; D-Ca-pantothenate, 0.05 mg; vitamin B_{12}, 10.0 µg; p-aminobenzoic acid, 0.05 mg; lipoic acid, 0.05 mg.

Bulk submission system at the DSMZ

The DSMZ welcomes bulk deposits (>10 isolates) (www.dsmz.de/bulk-deposit) of microbial strains isolated from host species or environmental habitats. Bulk deposits are time-intensive and take considerably longer to process than single strains. We therefore encourage depositors to start bulk deposits as early as possible in their projects to have them ready before the submission of accompanying manuscripts. Requests for urgent processing of bulk deposits during the revision phase of a manuscript cannot be considered.

Bulk deposits are processed in a standardised 3-step procedure.

1. Depositors are asked to fill out the Excel Bulk Deposit Accession Form covering all relevant strain information and sign the Bulk Deposit Agreement.

2. The coordinating curator requests the shipment of a first batch of up to 10 test strains for quality and legal checks as required for deposition in the DSMZ collection.

3. If all strains of step 2 pass these checks, the coordinating curator requests the remaining strains to be subsequently shipped in batches of 30-40 strains at a time. After the processing of each batch is completed, the next batch will be requested until the entire bulk deposit has been processed.

Depositors are strongly encouraged to request for integration of their strains into StrainInfo, which provides DOIs early in the deposition process (e.g., to use them in publications and sequence submissions). This enhances the traceability of the strains

throughout the deposition process. In this case the responsible curator can approve integration of a culture and its identity data into StrainInfo once the culture has been received by the DSMZ and passed initial checks. At this point the strain is assigned a DOI and is published on StrainInfo with the status “Deposition in progress”. Once the deposition process of the strain is completed the public status of the strain in the database is changed to “Published” with online catalogue status “available”.

Supplementary Results

Ecology of the pMMCAT megaplasmid

The matching pMMCAT plasmids were observed within species of *Bacteroides*, *Phocaeicola* and *Parabacteroides* from the USA, China, Japan, and Israel (**Supplementary Figure 1c**). Network analysis of the pMMCAT plasmids suggested those from *Phocaeicola* were more divergent than the *Bacteroides* and *Parabacteroides* pMMCAT plasmids (**Supplementary Figure 1d**). MobMess[15] analysis assigned pMMCAT_H253 as a compound plasmid, supporting the theory that it contains additional cargo, leading to the variable associations with health conditions for the predicted proteins. A sequence within the pMMCAT family was detected in 99.55% (1,774/1,782) of human gut metagenomes [15]. However, only 52 metagenomes had detection values >95%, including two samples from Fiji, with the highest coverage (135.82x) being from USA samples (**Supplementary Figure 1e**).

Separation of *Bacillota*

To validate the genomic tree generated using phylophlan, we created a second phylogenomic tree using the GTDB-Tk workflow (using commands: identify, align, infer) (**Supplementary Figure 4**). The GTDB-Tk inferred tree showed continual grouping of the *Bacillota*, however assignment of the isolates genomes by GTDB-Tk also suggested the separation of *Bacillota* into multiple phyla by the proposition of the separated genomes to "Bacillota_A". These results suggest inconsistency in the ability to phylogenomically place these taxa.

Variability in genome size and functionality between phyla

Genome size variation was observed between phyla, with *Actinomycetota* genomes being the smallest (2.38 ± 0.35 Mbp). *Bacteroidota* had the largest genomes (4.97 ± 1.02 Mbp), although not significantly larger than *Pseudomonadota* (4.84 ± 0.64 Mbp; adj. $p = 0.56$) (**Supplementary Figure 5a**; **Supplementary Data 3**). This variation in genome size between phyla was validated using >5,000 previously published genomes [1] (**Supplementary Figure 5b/c; Supplementary Data 3**). In addition to having the largest genomes, *Bacteroidota* also had the largest repertoire of CAZymes (**Supplementary Figure 6b**). To account for the variation in coding potential of each strain, and thereby examine their investment in carbohydrate utilisation, we normalised their CAZyme load based on their number of coding sequences (**Supplementary Figure 6c**). This analysis confirmed that the *Bacteroidota* have the highest investment in carbohydrate utilisation. However, they were followed by the *Actinomycetota* (**Supplementary Data 4**), indicating that, whilst members of *Actinomycetota* have undergone host-associated genome size reduction [2], they have invested in carbohydrate degradation.

16S rRNA gene sequence-based ecological analysis

To assess the occurrence of HiBC strains in the human gut, we looked at their prevalence and relative abundance within 1,000 human gut samples (**Supplementary Figure 7**). This analysis was complemented by screening the genome of each isolate against a collection of 42,927 MAGs [1,3]. *Bifidobacterium* spp. were some of the most prevalent (~70% of samples) and abundant (~9% on average) species. Cultivation and sequencing approaches capture different fractions of the human gut; this is highlighted by our ability to isolate a strain of *Selenomonas noxia*, which was only observed in a single sample, with a relative abundance <0.1%, making it a sub-dominant member of the gut microbiota, likely of oral origin [4]. Although only present at an average relative abundance of 2.7%, strains of *Agathobacter rectalis* (synonym *Eubacterium rectale*) were identified as being the most frequently reconstructed strain (n = 1,839 MAGs). In contrast, 19 strains did not match any MAGs, including seven novel taxa. To further assess the importance of HiBC species to the human gut ecosystem, we screened them against the original Human Microbiome Projects “most wanted” list of taxa [5]. Of the 1,468 most wanted sequences, 90 matched HiBC strains, including 4 high, 24 medium, and 62 low priority sequences. Of these, 8 matching strains represented novel taxa within the phylum *Bacillota* according to the current state of nomenclature, including two novel genera (**Supplementary Figure 8**). These “most wanted” species, and all other novel taxa discovered in this study are described in the main text at the end of the methods.

Effect of plasmids on ANI value calculation

Given the number of plasmids a single strain can contain, and the size a single plasmid can reach (see main results), we tested whether they could affect the taxonomic identification of an isolate, as most genomes lack delineation of plasmids. Given the promiscuous nature of plasmids [6], it is possible that the taxonomic signal of a strain may be masked by the presence of multiple large plasmids, distorting taxonomic assignment. As average nucleotide identity (ANI) is a standard approach for taxonomic assignment of genomes, we calculated the theoretical plasmid size required to potentially cause a >1% change in ANI between two genomes of identical size (**Supplementary Figure 9a**). Plasmids >80 kp should theoretically cause such a difference in ANI within genomes ≤8 Mb. To assess this within the HiBC strains, we selected all plasmid-containing strains that had a close relative (determined by GTDB-Tk) (n = 152). We then calculated the ANI to these close relatives, both with and without plasmid inclusion (**Supplementary Figure 9b**). The plasmids had a significant (p = 0.01), but negligible (delta-ANI, 0.004 ± 0.01) effect on reducing the ANI to the closest relative strains. The effect of plasmid inclusion on ANI was independent of cumulative plasmid size, with some small plasmid sets having a greater effect on ANI than the largest. The only strain on which the removal of plasmids had a significant effect was CLA-ER-H4, which was assigned to 'Collinsella sp900547855' by GTDB-

Tk when its plasmids were included, but was assigned to 'Collinsella sp003487125' without the plasmids. This is indicative of the need for better taxonomic study of this genus, as the plasmids had no effect on the assignment of CLA-ER-H4 to 'Collinsella sp003487125', but removing the plasmids increased ANI to 'Collinsella sp900547855' by 0.05%. However, to avoid the creation of false species within *Collinsella*, we have not described any new species within this genus, which needs to be done in a comprehensive manner.

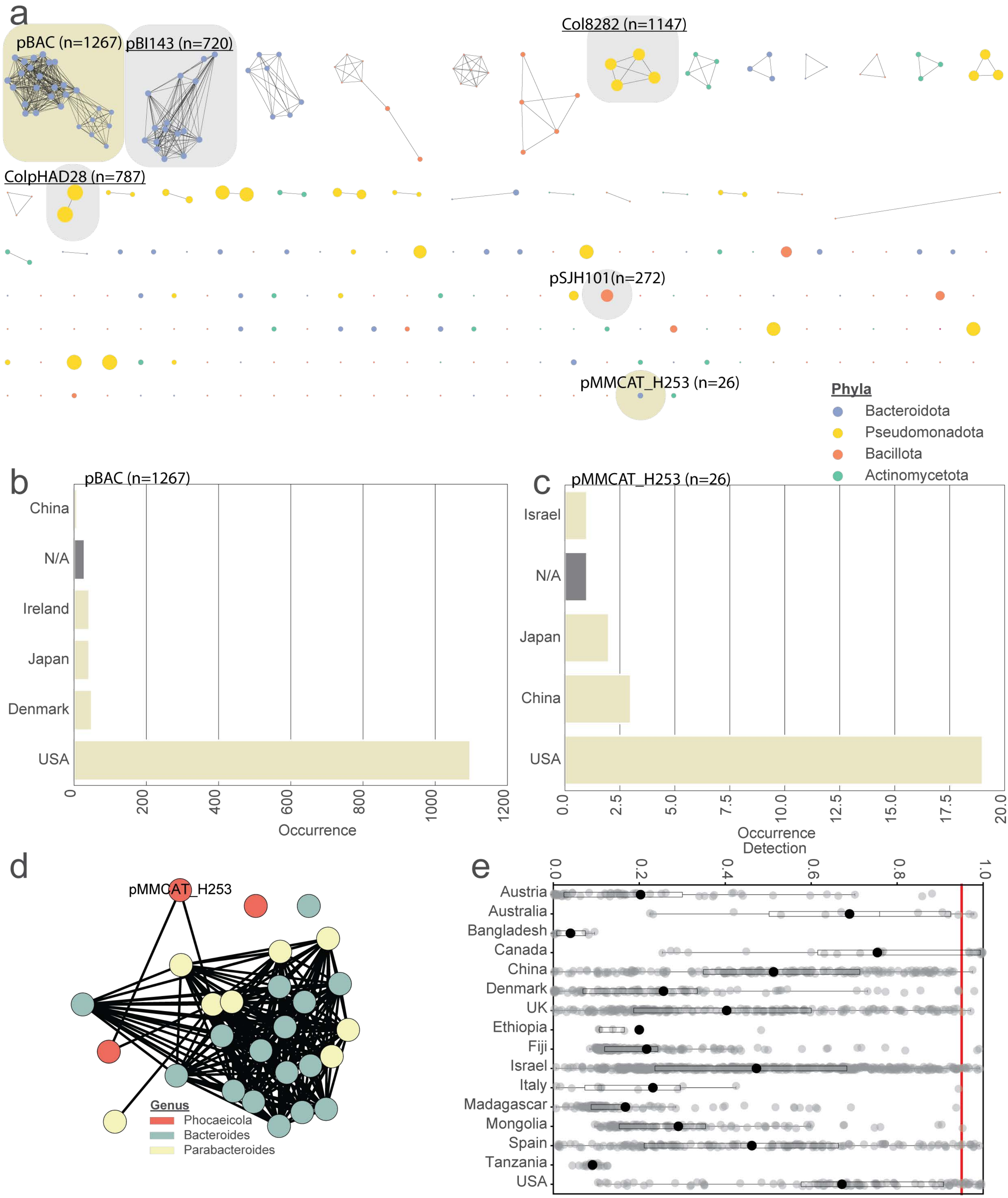

Supplementary Figure 1. a. Network visualisation of plasmid sequence similarity. Plasmids (circles) with similar sequences are connected with edges, the distance of which is proportional to their similarity (shorter = similar; longer = dissimilar). Plasmids are coloured based on their phyla and their size is proportional to the number of matching plasmids within PLSDB. Plasmids / Clusters of interest are named (underlined for known) and highlighted; grey for known, brown for novel. **b.** Country occurrence of the pBAC plasmid. **c.** Country occurrence of the pMMCAT_H253 plasmid. **d.** Mobmess network comparison of sequence similarity between pMMCAT_H253 matches within PLSDB. Each node represents a plasmid sequence, coloured based on the taxonomy of their originating isolate. The length of edges between nodes is based on the pairwise ANI comparison of plasmids. **e.** Detection of pMMCAT_H253 across 1,782 metagenomes, grouped based on their country of origin. Detection is defined as the proportion of nucleotodies within the pMMCAT reference sequence covered by atleast one read within the studied sample.

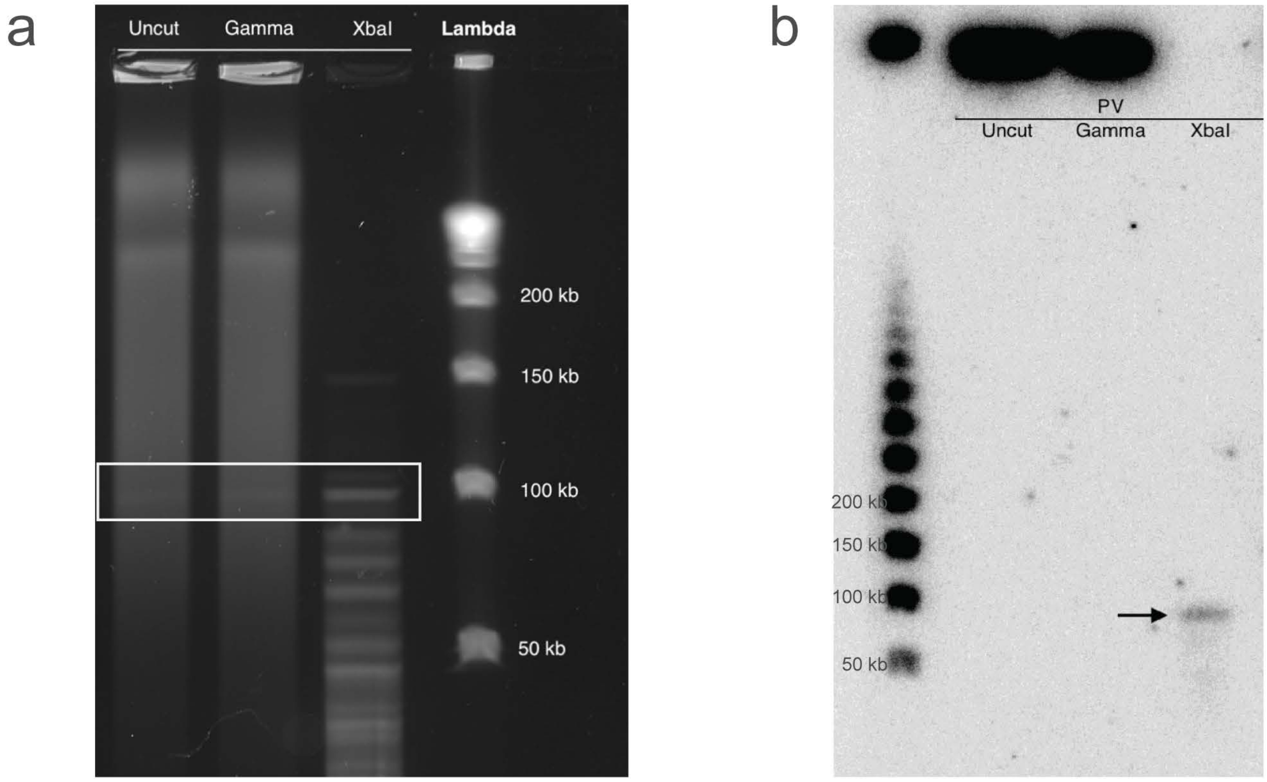

Supplementary Figure 2. a. Pulsed-field gel electrophoresis of pMMCAT_H253 confirms its presence in the uncut/native form, as well as occurring after gamma irradiation (to linearise circular DNA), and cut with XbaI (cuts once in pMMCAT_H253). Lambda phage was used as a marker size ladder. **b.** Southern blot a pulsed-field gel including the same groups. A single band appears in the XbaI lane just below the 100 kb ladder band, highlighted by an arrow.

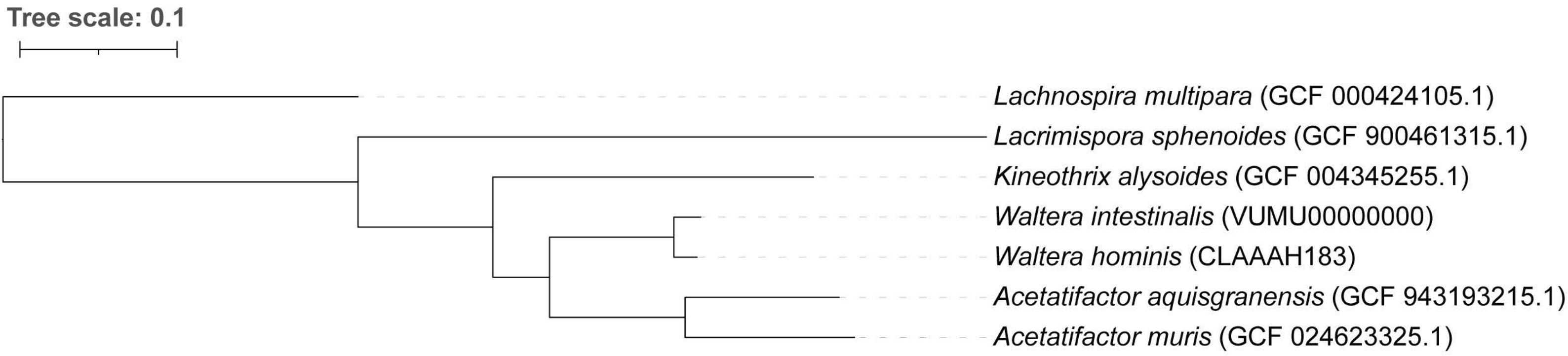

Supplementary Figure 3. Phylogenomic tree of both validly named *Acetatifactor*, and the only currently validly named *Waltera* species, with the proposed *Waltera hominis*. The type species for both *Kineothrix* and *Lacrimispora* are also included to provide greater context. The tree is rooted on the outgroup of *Lachnospira multipara*.

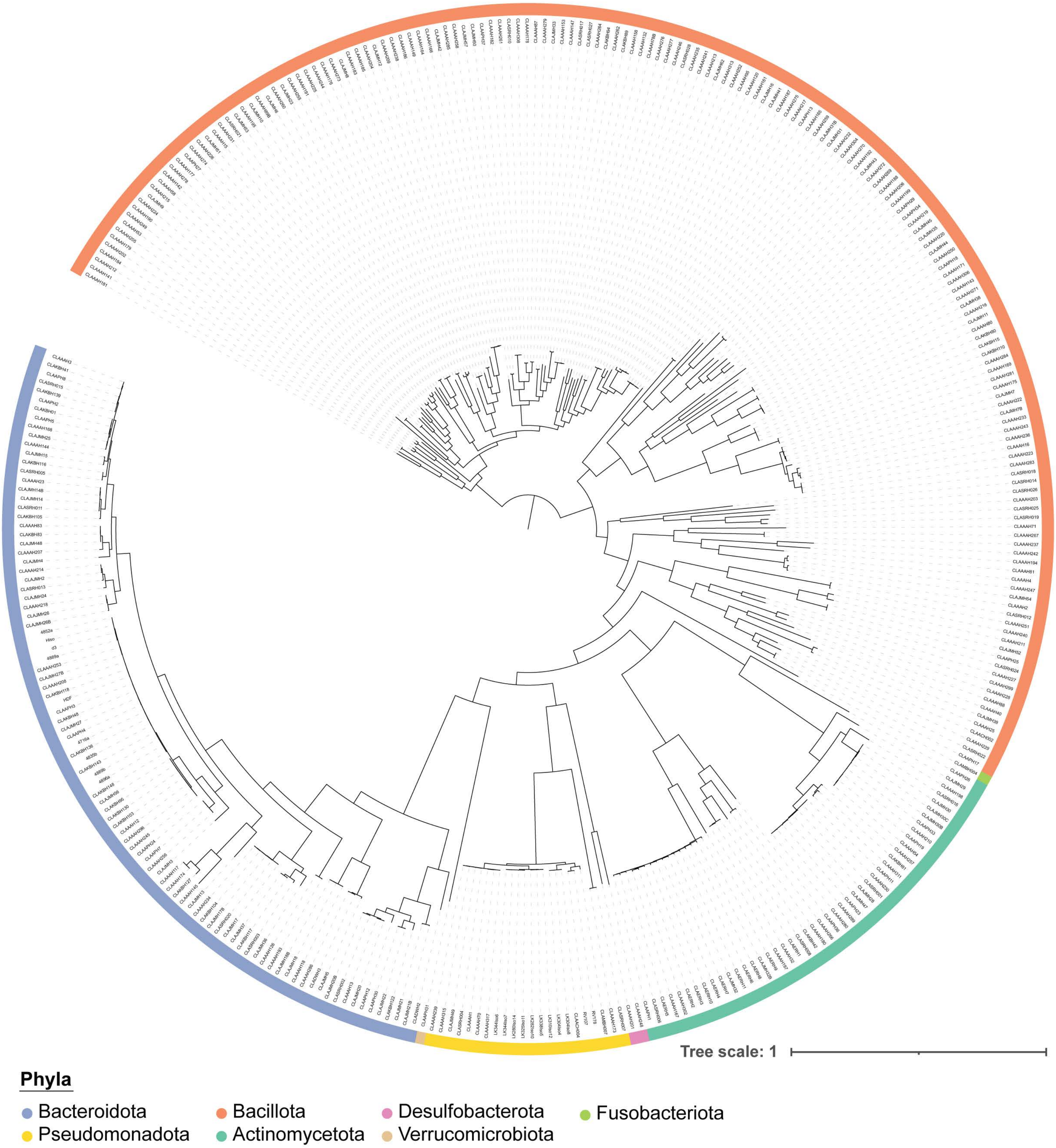

Supplementary Figure 4. Phylogenomic tree of the HiBC strains genomes created using GTDB-Tk. Each isolates assigned phyla is indicated by the colour on the outer ring.

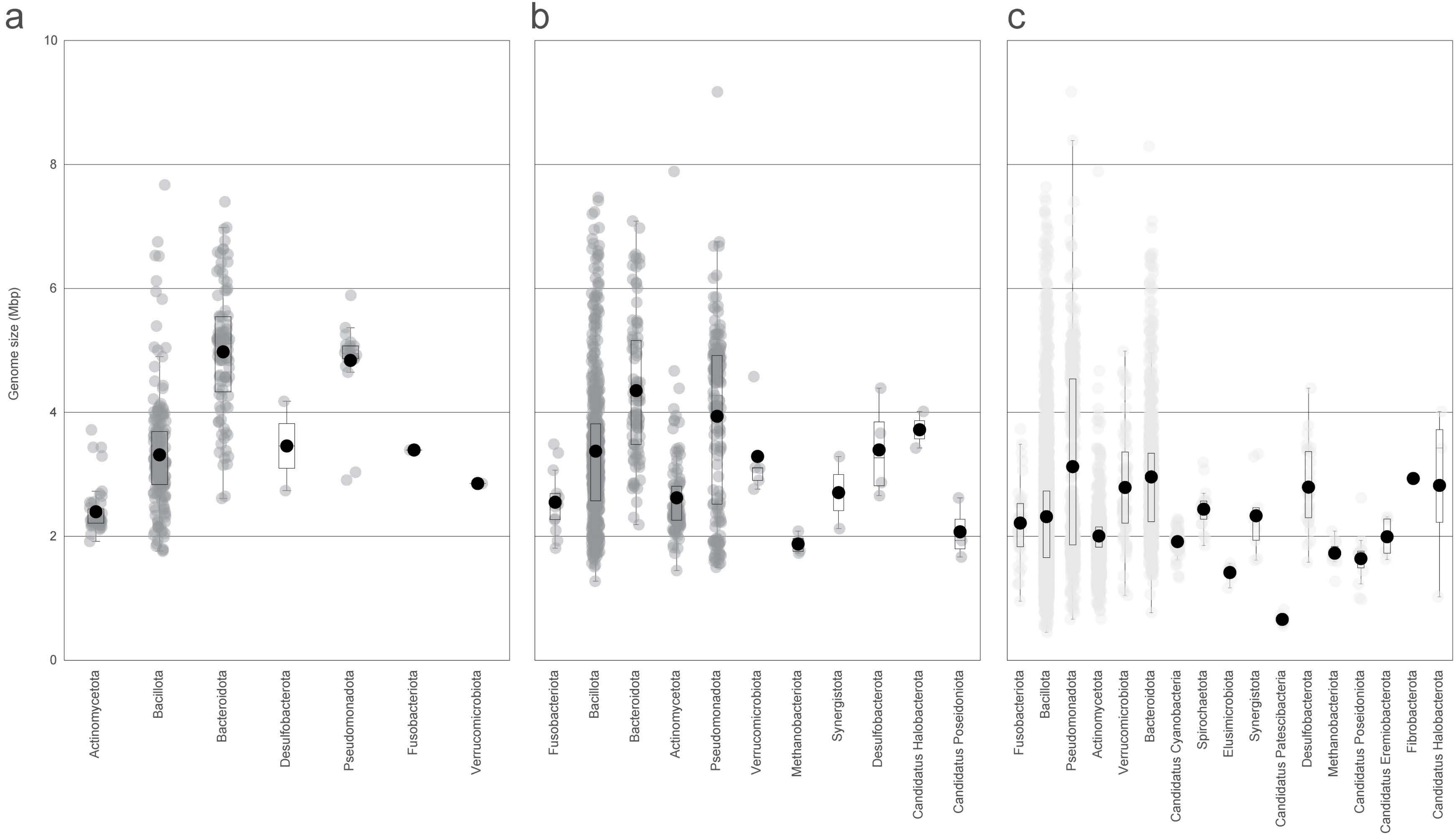

Supplementary Figure 5. a. The genome size of each HiBC strain (n = 340) was calculated in mega base pairs (Mbp). **b.** Genome size of all isolates (n = 891) within the UHGG collection. **c.** The genome size of all genomes (isolates and MAGs) within the UHGG collection (Almeida *et al*, 2021) (n = 4614). Black dots represent the mean value for each group. Statistical comparison of all groups is provided in Supplementary Table 1.

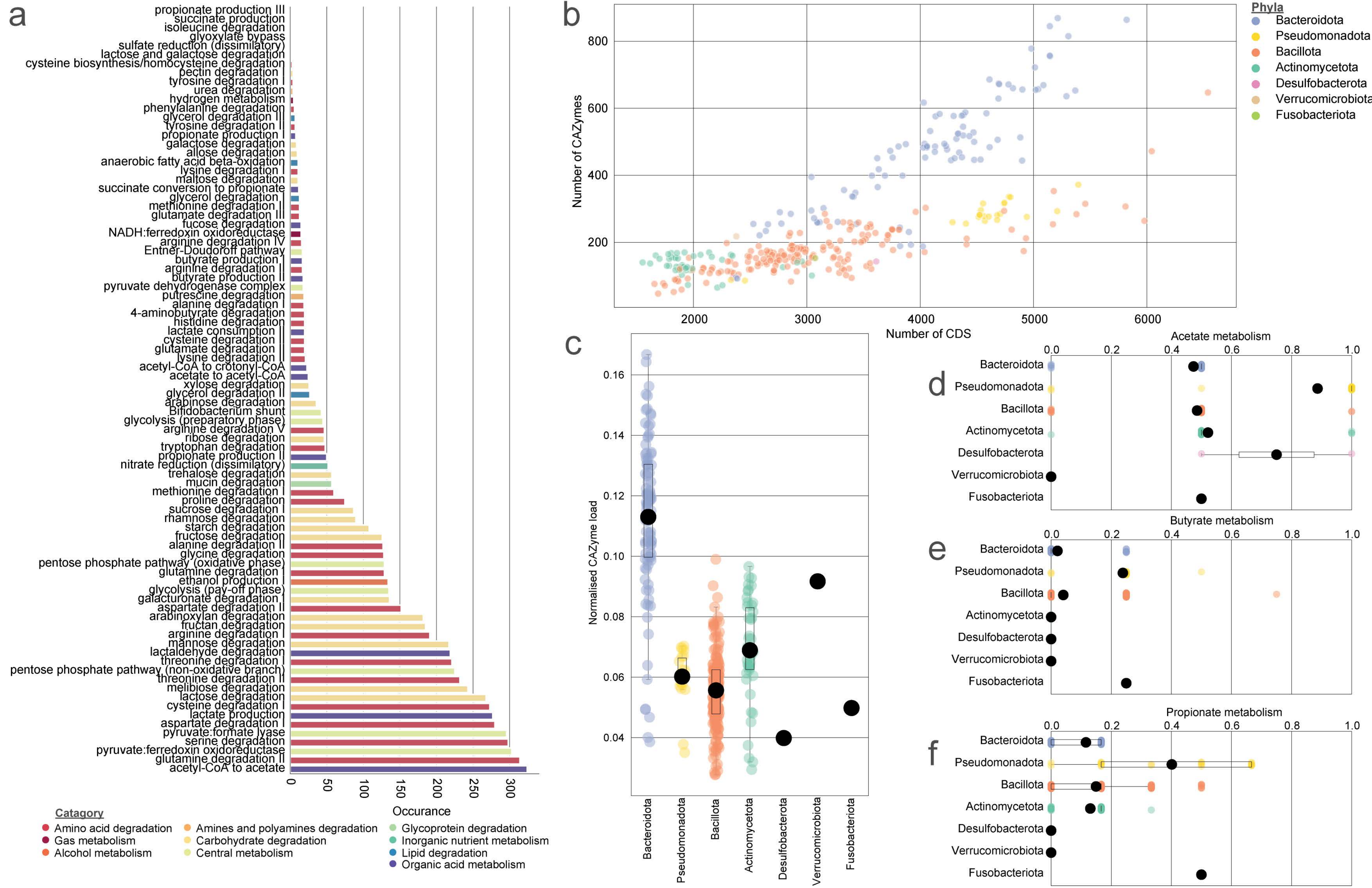

Supplementary Figure 6. a. Frequency of each gut metabolic modules detection across the 340 HiBC strains. Modules are coloured according to their broad functional assignments. **b.** Comparison of each strains CAZyme repertoire compared to its coding potential (total number of coding sequences), coloured based on phyla. **c.** The CAZyme repertoire of each strain normalised to their total coding potential, grouped by phyla. Black dots represent the mean value for each group. **d-f.** The functional investment of strains belonging to each phyla into the production of acetate, butyrate, and propionate.

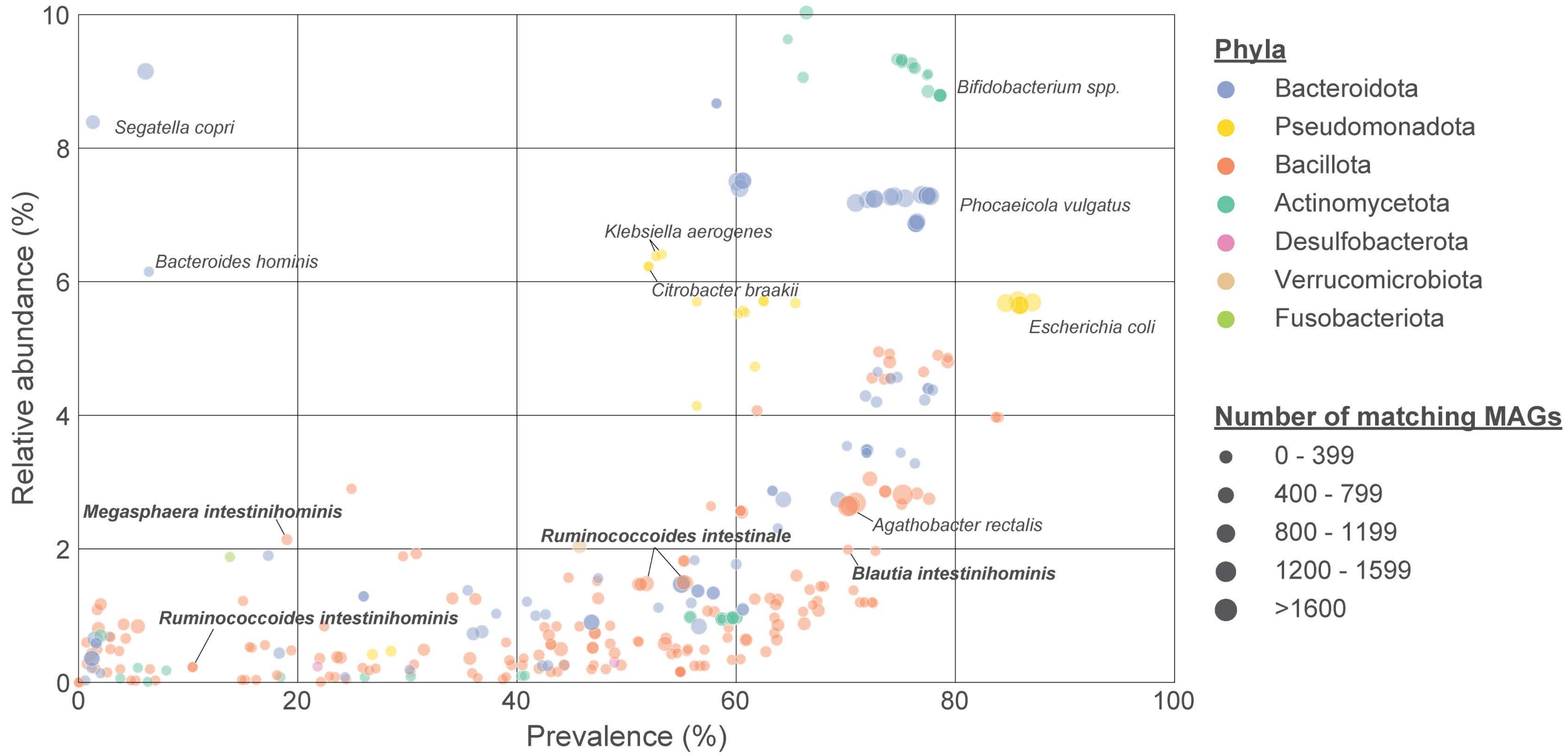

Supplementary Figure 7. The prevalence of each HiBC isolate across 16S rRNA amplicon datasets from the human gut (n = 1,000) was plotted against their relative abundance within positive samples. Each point represents a single isolate, coloured by phyla. The size of each point is proportional to the number of human gut MAGs matching the corresponding isolate's genome. A selection of interesting isolates discussed in this study, or taxonomically coherent isolates, are named, with novel taxa highlighted in bold.

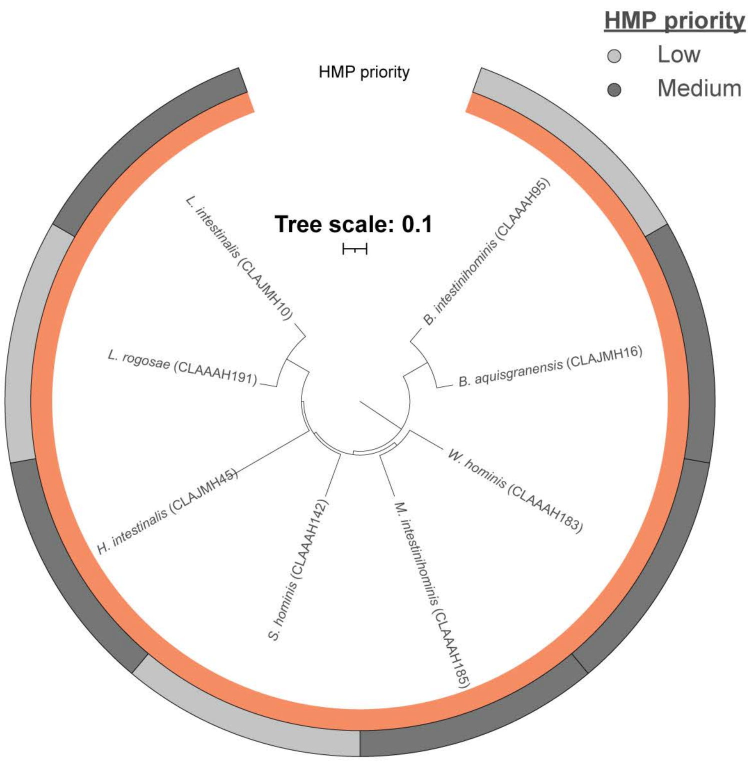

Supplementary Figure 8. Phylogenomic diversity of the eight novel species identified to be on the Human Microbiome Projects (HMP) most wanted listed. The inner ring is coloured according to the phyla assignment of the species, which are all *Bacillota*. The outer ring indicates the priority of each species in the most wanted list.

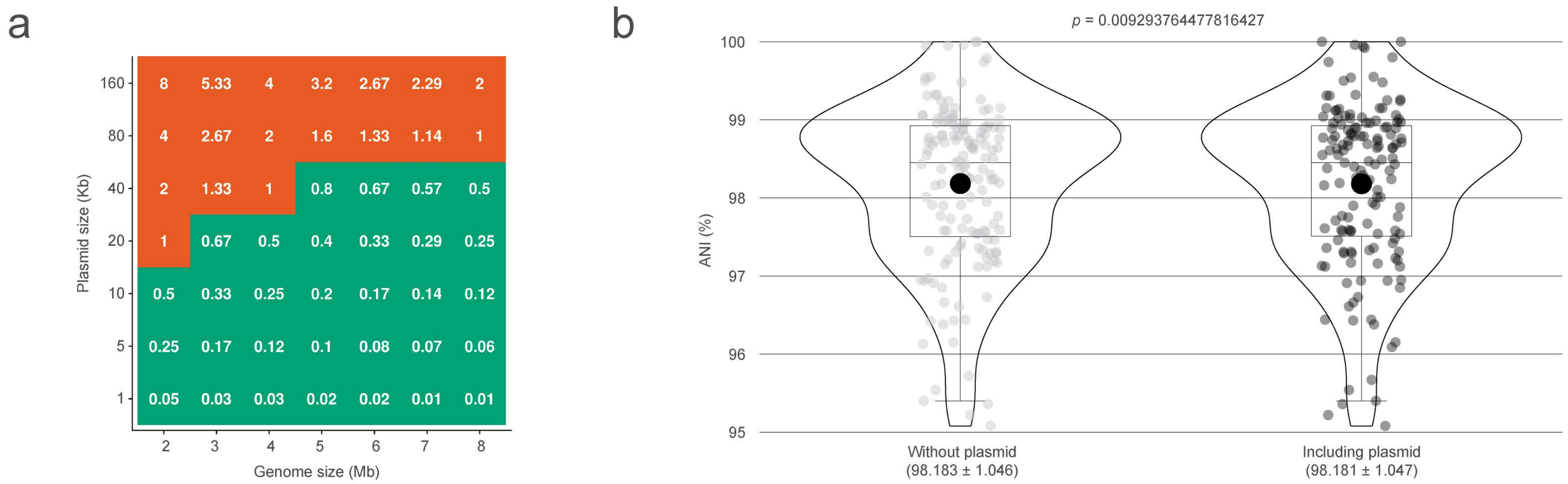

Supplementary Figure 9. a. The impact plasmids of specific sizes should theortically have on ANI comparison between two genomes of a set size was calculated and is stated in this matrix. In green are values less than 1%, when a plasmid has a >1% impact on ANI comparison, it is shown in orange. **b.** Comparison of each HiBC strain to its closest assignment, via GTDB-Tk, was calculated with and without plasmids (strains = 152). The presence of plasmids within the genomes significantly reduced the ANI score to the closest relatives, although the impact on ANI was 0.004 ± 0.01 and never altered the taxonomic assignment of a strain.

References

1. Almeida, A. *et al.* A unified catalog of 204,938 reference genomes from the human gut microbiome. *Nat Biotechnol* (2020) doi:10.1038/s41587-020-0603-3.
2. Seshadri, R. *et al.* Expanding the genomic encyclopedia of Actinobacteria with 824 isolate reference genomes. *Cell Genomics* **2**, (2022).
3. Pasolli, E. *et al.* Extensive Unexplored Human Microbiome Diversity Revealed by Over 150,000 Genomes from Metagenomes Spanning Age, Geography, and Lifestyle. *Cell* **176**, 649-662.e20 (2019).
4. Cruz, P., Mehretu, A. M., Buttner, M. P., Trice, T. & Howard, K. M. Development of a polymerase chain reaction assay for the rapid detection of the oral pathogenic bacterium , Selenomonas noxia. *BMC Oral Health* 1–8 (2015) doi:10.1186/s12903-015-0071-1.
5. Fodor, A. A. *et al.* The 'most wanted' taxa from the human microbiome for whole genome sequencing. *PLoS One* **7**, (2012).
6. Pujol, C., Ehrlich, S. D. & Jannière, L. The promiscuous plasmids pIP501 and pAMβ1 from gram-positive bacteria encode complementary resolution functions. *Plasmid* vol. 31 100–105 Preprint at https://doi.org/10.1006/plas.1994.1010 (1994).
