# Supplementary material for: HiBC: a publicly available collection of bacterial strains isolated from the human gut
Source: Nat Commun. 2025 May 6;16:4203. doi: 10.1038/s41467-025-59229-9 (PMC12056005; doi:10.1038/s41467-025-59229-9)
Supplement: Supplementary file 2 — Description of Additional Supplementary Files [file 41467_2025_59229_MOESM2_ESM.pdf]

Supplementary Data

Supplementary Data 1: List of existing isolate collections from the human gut. For each isolate collection we detail the accessibility of the published strains. Additional sheets provide specific information on strain collections

Supplementary Data 2: HPLC measurement of the production and/or utilisation of multiple metabolites.

Supplementary Data 3: Statistical comparison of genome sizes of phyla present within the HiBC, UHGG (isolates), and complete UHGG. Comparisons were conducted using a two-sided Wilcoxon rank-sum test, with p-value correction using Benjamini-Hochberg approach.

Supplementary Data 4: Statistical comparison of the normalised carbohydrate active enzyme investment of phyla within the HiBC. Comparisons were conducted using a two-sided Wilcoxon rank-sum test, with p-value correction using Benjamini-Hochberg approach.
