# Supplementary material for: HiBC: a publicly available collection of bacterial strains isolated from the human gut
Source: Nat Commun. 2025 May 6;16:4203. doi: 10.1038/s41467-025-59229-9 (PMC12056005; doi:10.1038/s41467-025-59229-9)
Supplement: Supplementary file 7 — Reporting Summary [file 41467_2025_59229_MOESM7_ESM.pdf]

nature portfolio

Corresponding author(s): Thomas Clavel

Last updated by author(s): Jul 2, 2024

Reporting Summary

Nature Portfolio wishes to improve the reproducibility of the work that we publish. This form provides structure for consistency and transparency in reporting. For further information on Nature Portfolio policies, see our Editorial Policies and the Editorial Policy Checklist.

Statistics

For all statistical analyses, confirm that the following items are present in the figure legend, table legend, main text, or Methods section.

n/a	Confirmed	
☐	☒	The exact sample size (*n*) for each experimental group/condition, given as a discrete number and unit of measurement
☐	☒	A statement on whether measurements were taken from distinct samples or whether the same sample was measured repeatedly
☐	☒	The statistical test(s) used AND whether they are one- or two-sided<br>*Only common tests should be described solely by name; describe more complex techniques in the Methods section.*
☒	☐	A description of all covariates tested
☐	☒	A description of any assumptions or corrections, such as tests of normality and adjustment for multiple comparisons
☐	☒	A full description of the statistical parameters including central tendency (e.g. means) or other basic estimates (e.g. regression coefficient) AND variation (e.g. standard deviation) or associated estimates of uncertainty (e.g. confidence intervals)
☐	☒	For null hypothesis testing, the test statistic (e.g. *F*, *t*, *r*) with confidence intervals, effect sizes, degrees of freedom and *P* value noted<br>*Give P values as exact values whenever suitable.*
☒	☐	For Bayesian analysis, information on the choice of priors and Markov chain Monte Carlo settings
☐	☒	For hierarchical and complex designs, identification of the appropriate level for tests and full reporting of outcomes
☒	☐	Estimates of effect sizes (e.g. Cohen's *d*, Pearson's *r*), indicating how they were calculated

Our web collection on statistics for biologists contains articles on many of the points above.

Software and code

Policy information about availability of computer code

Data collection	https://github.com/ClavelLab/genome-assembly; https://git.rwth-aachen.de/clavellab/hibc
Data analysis	Trimmomatic (v0.39); BBtools; plasmidSPades (v3.15.5); Recycler (v0.7); SPades (v3.15.5); CheckM (v1.2.2); QUAST (v5.0.2) ; metaxa2 (v2.2.3) ; bakta (v1.6.1) ; Snakemake (v7.9.0) ; Protologger (v1.3) ; barrnap (v0.9) ; Phylophlan v3.0.60 ; Kofamscan v1.3.0 ; FastANI v1.34; IMNGS ; BLASTN v2.9.0+ ; MobMess ; PLSDB (v2023_11_03_v2) ; Easyfig v2.2.2 ; Rotate v1.0 ; AlphaFoldServer v3 ; GTDB-Tk r214

For manuscripts utilizing custom algorithms or software that are central to the research but not yet described in published literature, software must be made available to editors and reviewers. We strongly encourage code deposition in a community repository (e.g. GitHub). See the Nature Portfolio guidelines for submitting code & software for further information.

Data

Policy information about availability of data

All manuscripts must include a data availability statement. This statement should provide the following information, where applicable:

- Accession codes, unique identifiers, or web links for publicly available datasets
- A description of any restrictions on data availability
- For clinical datasets or third party data, please ensure that the statement adheres to our policy

The genomes for all strains have been deposited at NCBI under BioProject: PRJNA996881. Bulk download of HiBC resources is possible via Zenodo for the genomes (https://doi.org/10.5281/zenodo.12180083), plasmid sequences (https://doi.org/10.5281/zenodo.12187897), 16S rRNA gene sequences (https://doi.org/10.5281/zenodo.12180259) and the isolates metadata (https://doi.org/10.5281/zenodo.12180506). The PacBio genome for P. vulgatus CLA-AA-H253 (=DSM 118718) has

been deposited in ENA under accession code PRJEB80480 [https://www.ncbi.nlm.nih.gov/bioproject/ PRJEB80480] and Zenodo [https://doi.org/10.5281/zenodo.14674027].

Research involving human participants, their data, or biological material

Policy information about studies with human participants or human data. See also policy information about sex, gender (identity/presentation), and sexual orientation and race, ethnicity and racism.

Reporting on sex and gender	We confirm that we used the term sex (male or female) carefully to define the donors of stool material for the isolation of bacteria
Reporting on race, ethnicity, or other socially relevant groupings	Not relevant
Population characteristics	Human adult subjects that donated stool samples
Recruitment	Mailing lists of local research consortia
Ethics oversight	The Ethics Committee of the Medical Faculty of RWTH University Aachen permitted bacterial isolation from human stool under ethical number EK 23-055, EK 316-16, and EK 194/19. For strains originating from Vienna, isolation was approved by the University of Vienna ethics committee under ethical number 00161. For strains originating from Braunschweig, isolation was approved by the Ethics Committee of Lower Saxony (MHH permit No. 6794, 8629, and 8750). Written informed consent was signed by all enrolled participants.

Note that full information on the approval of the study protocol must also be provided in the manuscript.

Field-specific reporting

Please select the one below that is the best fit for your research. If you are not sure, read the appropriate sections before making your selection.

☒ Life sciences ☐ Behavioural & social sciences ☐ Ecological, evolutionary & environmental sciences

For a reference copy of the document with all sections, see nature.com/documents/nr-reporting-summary-flat.pdf

Life sciences study design

All studies must disclose on these points even when the disclosure is negative.

Sample size	Large-scale (>1,000 public datasets) for bioinformatic analysis; triplicate cultures for metabolite production
Data exclusions	No data was excluded
Replication	All bacterial strains were made publicly available to enable replication. Work with plasmids and to test butyrate production included a sufficient number of replicates and was repeated in independent experiments.
Randomization	Not relevant
Blinding	The work did not include group allocation that required blinding. Comparison of the occurrence of bacteria or their proteins in health and disease included metadata from published studies that were analysed bioinformatically without blinding.

Reporting for specific materials, systems and methods

We require information from authors about some types of materials, experimental systems and methods used in many studies. Here, indicate whether each material, system or method listed is relevant to your study. If you are not sure if a list item applies to your research, read the appropriate section before selecting a response.

Materials & experimental systems

n/a	Involved in the study	
☒	☐	Antibodies
☒	☐	Eukaryotic cell lines
☒	☐	Palaeontology and archaeology
☒	☐	Animals and other organisms
☒	☐	Clinical data
☒	☐	Dual use research of concern
☒	☐	Plants

Methods

n/a	Involved in the study	
☒	☐	ChIP-seq
☒	☐	Flow cytometry
☒	☐	MRI-based neuroimaging

Plants

Seed stocks	Not relevant
Novel plant genotypes	Not relevant
Authentication	Not relevant
